# Expression of Constitutive Fusion of Ubiquitin to PCNA Restores the Level of Immunoglobulin A/T Mutations During Somatic Hypermutation in the Ramos Cell Line

**DOI:** 10.3389/fimmu.2022.871766

**Published:** 2022-04-01

**Authors:** Leticia K. Lerner, Dorine Bonte, Morwenna Le Guillou, Mahwish Mian Mohammad, Zeinab Kasraian, Alain Sarasin, Emmanuelle Despras, Said Aoufouchi

**Affiliations:** ^1^ Centre National de la Recherche Scientifique UMR 9019, B Cell and Genome Plasticity Team, Villejuif, France; ^2^ Gustave Roussy, Villejuif, France; ^3^ Université Paris-Saclay, Orsay, France; ^4^ Department of Microbiology, Institute of Biomedical Sciences, University of São Paulo, São Paulo, Brazil; ^5^ Sorbonne Université, Paris, France

**Keywords:** immunoglobulin somatic hypermutation, PCNA monoubiquitination, Ramos B cell line, USP1 inhibition, A/T mutation pathway

## Abstract

Somatic hypermutation (SHM) of immunoglobulin (Ig) genes is a B cell specific process required for the generation of specific and high affinity antibodies during the maturation of the immune response against foreign antigens. This process depends on the activity of both activation-induced cytidine deaminase (AID) and several DNA repair factors. AID-dependent SHM creates the full spectrum of mutations in Ig variable (V) regions equally distributed at G/C and A/T bases. In most mammalian cells, deamination of deoxycytidine into uracil during S phase induces targeted G/C mutagenesis using either direct replication of uracils or TLS mediated bypass, however only the machinery of activated B lymphocytes can generate A/T mutagenesis around AID-created uracils. The molecular mechanism behind the latter remains incompletely understood to date. However, the lack of a cellular model that reproduces both G/C and A/T mutation spectra constitutes the major hurdle to elucidating it. The few available B cell lines used thus far to study Ig SHM indeed undergo mainly G/C mutations, that make them inappropriate or of limited use. In this report, we show that in the Ramos cell line that undergoes constitutive G/C-biased SHM in culture, the low rate of A/T mutations is due to an imbalance in the ubiquitination/deubiquitination reaction of PCNA, with the deubiquitination reaction being predominant. The inhibition of the deubiquitinase complex USP1-UAF1 or the expression of constitutive fusion of ubiquitin to PCNA provides the missing clue required for DNA polymerase η recruitment and thereafter the introduction of A/T base pair (bp) mutations during the process of IgV gene diversification. This study reports the establishment of the first modified human B cell line that recapitulates the mechanism of SHM of Ig genes *in vitro*.

## Introduction

The process of SHM in germinal center (GC) B cells is basically the result of two distinct molecular mechanisms taking place in separate phases of the cell cycle (1). Both mechanisms start with the introduction of uracils along the region that is subject to SHM in immunoglobulin (Ig) genes by AID-dependent deamination of deoxycytidine residues. When this occurs during S phase of the cell cycle, this leads to mutations mainly focused on dC/dG pairs creating both transitions and transversions. This mutagenic process involves either the action of uracil DNA glycosylase (UNG), which creates an abasic site by removing the uracil that is subsequently bypassed by translesion DNA polymerases (TLS pols), or through the direct copy of the deoxy-uracil (dU) by the replicative DNA polymerases (2). These dU could be either generated during S phase or as shown recently generated during G1 of the cell cycle and survive until S/G2 due to the activity of Fam72a that reduces UNG levels in G1 (3, 4). Conversely, the mutations are spread on the surrounding A/T bases using an error-prone gap filling reaction by DNA polη (5–7). This latter is initiated by MSH2/MSH6 recognition of the dU:dG mismatch, followed by the action of exonuclease 1 (EXO 1), which creates the single-stranded gap in a process called noncanonical mismatch repair (ncMMR) (8–10). Furthermore, the monoubiquitination of proliferating cell nuclear antigen (mUb-PCNA) is a major posttranslational modification (PTM) required for the generation of mutations at A/T base pairs during SHM (11). PCNA undergoes monoubiquitination (mUb) mainly in response to replication fork stalling (12). This PTM orchestrates polymerase switching that favors the recruitment of TLS polymerases for lesion-bypass DNA synthesis during replication (13). However, in the case of SHM of Ig genes, how PCNA is monoubiquitinated in G1 in the absence of DNA lesions and how it participates in the specific recruitment of polη remain unknown. PCNA that forms the eukaryotic DNA sliding clamp is an auxiliary factor of DNA polymerases. Active PCNA is composed of the association of three monomers in a ring-shaped structure (14). In mammalian cells, to ensure cell survival, PCNA is modified at a conserved site, K164, *via* a single ubiquitin polypeptide moiety by RAD6 and RAD18 in response to various DNA damaging agents (15). When the lesion is bypassed, the ubiquitin polypeptide is removed mainly by ubiquitin-specific protease 1 (USP1) to allow the recruitment of high-fidelity DNA polymerases and resume replication. Previous work from H. Jacobs’s group (The Netherlands Cancer Institute, Amsterdam) has shown that a mouse expressing PCNA with a lysine-to-arginine mutation at residue 164 preventing the mUb displays a phenotype similar to polymerase η and mismatch repair-deficient B cells with a strong reduction of somatic mutations at A/T bases in Ig V region (IgV) associated with a compensatory increase at G/C mutations (11). A similar result was obtained using knockout mice for PCNA expressing exogenous PCNA with the K164R mutation (16). However, in activated B lymphocytes, the situation is particular since the PMT of PCNA takes place in the absence of undamaged DNA *via* an unknown mechanism, and the main goal is to generate mutations (17).

The search for lymphoid cell lines that could provide a tractable system for investigating *in vitro* the process of SHM in general and, more specifically, the process of A/T mutagenesis in particular started several decades ago (18–22). Many mouse and human B cell lines have been identified. Among them, the human Burkitt lymphoma cell lines CL-O1, BL2 and Ramos have been extensively studied. These cells were transformed in the germinal center (GC) during the process of antibody affinity maturation (23). Induction of somatic mutations in CL-01 cells requires cross-linking of the BCR and T cell contact through CD40/CD40 ligand and CD80/CD28 co-engagement. The BL2 cell line undergoes V_H_ diversification on culture in the presence of an anti-immunoglobulin and coculture with activated T cells (18) or through simultaneous aggregation of three surface receptors, IgM, CD19 and CD21 (21, 22). Ramos cells, however, diversify the IgV domain constitutively during culture (19, 20, 24). IgV gene diversification in both cell lines exhibits the major hallmarks of *in vivo* Ig SHM: the mutations are (I) largely base substitutions (II) targeted to transcribed V genes and especially concentrated at selected hotspot motifs RGYW/WRCY (R: purines, Y: pyrimidines and W: A or T) (III) dependent on AID activity and (IV) biased for transitions over transversions. However, despite the presence of intact components of the Ig A/T mutational machinery (24), the major drawback of these cells remains their inability to efficiently perform A/T mutagenesis. Therefore, they display a mutation pattern biased toward G/C mutations (80 to 90%), thus greatly limiting their use for elucidating the mechanism of A/T mutagenesis. In this report, we discovered that in Ramos cells, the paucity of A/T mutations is due to an imbalance in the ubiquitination and deubiquitination of PCNA, the latter being predominant. The inhibition of the deubiquitinase responsible or the expression of a constitutive fusion of ubiquitin to PCNA significantly increases the rate of A/T mutations, thus reviving the SHM A/T mutagenesis pathway and consequently providing the first *in vitro* system that can be used to elucidate the A/T mutagenesis process.

## Material And Methods

### Plasmids, Plasmid Construction and Cell Transfection

The His7-Ub-PCNA-K164R dsDNA fragment was synthesized by Eurofins Genomics. To avoid USP1-dependent deubiquitination, the C-terminal Gly codon of the ubiquitin gene was replaced by Arg, and PCNA Lys164 was replaced by Arg to avoid endogenous ubiquitination. The synthetized open reading frame was cloned into the plasmid vector pcDNA3.1.puro (Thermo Fisher) to make an N-terminal (mUb-PCNA-) fusion protein. pcDNA.3.1.zeo (Thermo Fisher) expressing full-length human pol eta full-length dsDNA was previously described (25). The pIRES-Hygro2 vector (Clontech, Palo Alto, CA) expressing full-length human AID was a gift from CA Reynaud (21). Ramos cells were transfected with the desired plasmid by electroporation (Amaxa) according to the manufacturer’s protocol. Stably transfected clones were selected with the appropriate antibiotic. Stable transfectants were isolated and further propagated in medium containing 600 ng/mL puromycin (*In vivo*Gen) for the cells expressing His-mUb-PCNA, 150 μg/mL Zeocin (*In vivo*Gen) for clones expressing exogenous POLH or 500 μg/mL hygromycin (Roche, Mannheim, Germany) for clones expressing exogenous AID.

### Cell Lines and Culture Conditions

We cultured Ramos and both Burkitt lymphoma and mantle cell lymphoma cells in RPMI 1640 +GlutaMAX medium (Gibco) supplemented with 10% fetal calf serum (FCS) and penicillin/streptomycin (Invitrogen). The non-B cells were cultured as reported in the literature. Human tonsils were obtained as discarded material from routine tonsillectomies. B cell isolation was performed as described in (26).

### Cell Treatments

For H2O2 treatment, cells were exposed to 1 mM H2O2 (Sigma–Aldrich) for 20 min at 37°C in MEM without FCS. After treatment, the cells were washed once with PBS and incubated in complete medium prior to harvesting. For UVC light treatment, the culture medium was removed, and cells in a dish were exposed to 254-nm UV irradiation at a dose of 10 J/m^2^. The culture medium was immediately added, and the cells were returned to incubation.

For immunoblotting, cells were collected 2 h later, and total protein was extracted and analyzed using SDS–PAGE and western blot.

### Inactivation of the *POLH* and *AID* Genes in Ramos Cells

For gene deletion, a pair of single guide RNAs (sgRNAs) were designed with the CRISPOR program. One plasmid expressing both gRNA, Cas9 and green fluorescent protein (LentiCRISPRV2GFP, Plasmid # 82416 Addgene) was nucleofected into Ramos cells using Amaxa (Lozano) according to the manufacturer’s protocol. At 24 h after transfection, GFP cells were sorted with BD FCAS Aria II and plated into single clones in 96-well plates. Individual clones were genotyped by PCR to identify mutated clones by insertion or deletion. Candidate clones were further confirmed by Sanger sequencing and western blot. Guide RNA sequences: pol *eta* gRNAfor: 5’-GGTGAGGTTAGCTTTCCCAC-3’ and pol *eta* gRNARev: 5’-GTGGGAAAGCTAACCT-CACC-3’, AICDA gRNAfor: 5’-GTGGAATTGCTCTTCCTCC-3’, AICDA gRNARev: 5’-GGAGGAAGAG CAATTCCAC-3’. A vector expressing full-length human polη was described previously (27), and a vector expressing full-length human AICDA was described in (21) and used for complementation of the KO cell lines. polη Zeocin- and AID puromycin-resistant clones were selected with 150 μg/mL Zeocin (Roche, Mannheim, Germany) and 600 ng/mL puromycin (Invitrogen), respectively.

### Analysis of SHM in Ramos Cells

Genomic DNA was isolated after 42 days of culture and cell sorting. The rearranged V_H4_DJ_H6_ region was amplified with two rounds of PCR using Phusion DNA polymerase (Thermo Fisher Scientific), the primers Vh4.1 for 5′- CAGGTGCAGCTACAGCAG -3′ and Jh6.1Rev 5′- GCTGA- GGAGACGGTGACC -3′ for the first round and the primers Vh4.2 for 5′- TGGGGCGCAGGACTGTTGAA -3′ and Jh6.2 Rev 5′- GACCGTGGTCCCTTGGCC -3′, for the second round. The conditions for the first PCR amplification were 98°C for 2 min, 20 cycles at 98°C for 10 s, 70°C for 20 s and 72°C for 20 s, and for the second PCR, 30 cycles at 98°C for 10 s and 72°C for 30 s. For amplification of the constant Cmu 2-4 region, we used the primers Cmu. for 5’- CGGACCAGGTGCAGGCTGAGGCC -3’ and Cmu. Rev 5’- CTCCCGCAGGTTCAG CTGCTCCC -3’ with the following program 98°C for 2 min, 35 cycles at 98°C for 10 s and 72°C for 20 s. The PCR products were gel-purified with a QIAquick gel extraction kit (Qiagen, Hilden, Germany) and cloned with CloneJET PCR cloning kit (ThermoFisher scientific). Plasmid DNA extracted from individual bacterial colonies and sequencing using Sanger sequencing were performed by Eurofins Genomics.

### Flow Cytometry and Sorting

Cells were collected and labeled with anti-human IgM-FITC antibodies (Ref# 31575; Invitogen) at 4°C for 20 min and then washed with PBS/1% BSA. To estimate the percentage of IgM-negative cells, FACS analyses were performed using a BD Accuri C6 flow cytometer (BD Biosciences). Cell sorting of IgM-negative cells was performed using a FACSAria III or Influx (BD Biosciences).

### Proliferation and Cell Cycle Analyses

Approximately 2x10^6^ cells were pelleted by centrifugation at 1200 rpm for 5 min. After Centrifugation, cells were washed in cold PBS and resuspended in PBS. The suspended cells were transferred dropwise into 4.5 mL of 70% ethanol and then fixed overnight at 4°C. The ethanol-suspended cells were then collected, washed and resuspended in 50 mg/mL propidium iodide (Sigma, P 4170)/0.1% (v/v) Triton X-100 staining solution with 100 µg/mL RNase A in the dark for 1 h at 37°C. A BD Accuri C6 flow cytometer (BD Biosciences) was used for analysis of cells. For cell proliferation, at day 0, 10^4^ viable cells were seeded in 48 plates in 200 µL of complete medium and incubated at 37°C and 5% CO2. Cells were counted at 24, 48, 72 and 96 h in the presence of Trypan blue using a Countess II FL automated cell counter (Life Technologies). All experiments were done in triplicates.

### Western Blotting and Cellular Fractionation

Samples were collected and placed on ice in a lysis solution [50 mM Tris–HCl (pH 8.0), 150 mM NaCl, 1 mM EDTA, 1% NP-40, 10% glycerol] containing 0.5% SDS and 2 mM PMSF with a protease inhibitor cocktail (Sigma P-8340, 1:100). Cellular proteins were resolved on a 12.5% SDS–PAGE gel. The membrane was incubated for 1 h at room temperature in 5% skim milk in PBS with 0.05% Tween-20 (PBST), and the membrane was probed with anti-PCNA PC10 (Ref # sc56; Santa Cruz), anti-alpha tubulin (Ref# MA1-80017; Thermo Fisher Scientific), anti-actin (Ref #MA1-744; Thermo Fisher Scientific), anti-Vinculin (clone 7F9, Ref# 14-9777-80; eBioscience), anti-AID (Ref #14-959-82; Thermo Fisher Scientific), anti-polη (Ref# ab17725; Abcam), anti-FancD2 (Ref# sc20022; Santa Cruz), anti-USP1 (Ref # ab108104Ref; Abcam), anti-Msh2 (Ref #A300-451A; Bethyl), and anti-Msh6 (Ref # A300-022A; Bethyl) antibodies. Immunoreactivity was detected using a horseradish peroxidase-conjugated secondary antibody.

### Nickel Beads Pull-Down

Whole-cell extracts were prepared in lysis buffer without EDTA supplemented with benzonase. The extracted proteins were adjusted to 20 mM imidazole and incubated with Ni-NTA-agarose (Qiagen) overnight at 4°C. Beads were then washed three times with the same lysis buffer without EDTA and containing 30 mM imidazole. Following the last wash, the beads were resuspended in 2X Laemmli buffer and boiled at 98°C for 5 min. The bound proteins were analyzed by immunoblotting using the indicated antibody.

## Results

### Low PCNA Monoubiquitination in Ramos Cells After Treatment With Genotoxic Agents Correlates With a High Amount of USP1 Deubiquitinase

**Figure 1:**
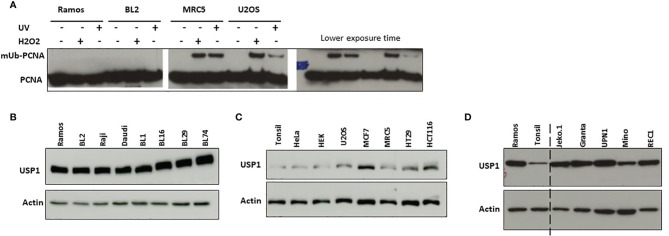
Induction of monoubiquitination of PCNA and USP1 expression in Ramos and BL2 cells. **(A)** B cell lines Ramos and BL2 cells (left pannel) and non-B cells MRC5 and U2OS cells (right pannel) were exposed to UVC light (10 J/m^2^) or incubated in the presence of H_2_O_2_ as indicated in the M&M. Total protein extracts were prepared 2 hours post-treatment, separated by SDS–PAGE and analyzed by immunoblotting using an anti-PCNA antibody. **(B, C)** Protein extracts were prepared from the indicated cell lines and analyzed as described above using an anti-USP1 antibody. In B–D actin was used as loading control. **(D)** Protein extracts from Ramos and Tonsillar B cells were loaded side by side to facilitate comparison.

Ramos cells lines, like most Burkitt lymphoma cell lines that undergo SHM in culture, display a strong bias in favor of mutations at G/C over A/T (18, 19, 28, 29). To date the reasons of such bias remain unknown. During SHM, A/T mutation induction requires, on the one hand, the activity of several factors, including AID, DNA mismatch repair proteins (MSH2/MSH6), polη and, on the other hand, the monoubiquitination of PCNA. We and others have shown previously that Ramos cells express unmutated full-length cDNA of *AID, UNG, POLH, PCNA, MSH2, MSH6* and *EXO1*. In addition, the cells are both BER and MMR proficient (22, 24, 30). Thus, there were no mutations, no lack of expression and no obvious evidence of dysfunction of any of these factors in Ramos cells. On the other hand, mUb of PCNA at the conserved K164 site is necessary for the recruitment of polη (11, 16), which is the sole mutator of A/T bases in the normal physiological context during SHM (7). We therefore asked whether the ubiquitination pathway of PCNA is deregulated in these cells. In mammalian cells, PCNA is monoubiquitinated by RAD6 and the RAD18 ubiquitin ligase complex in response to UV irradiation or other genotoxic agents; therefore, we used this property to investigate the induction of mUb-PCNA in Ramos cells under these conditions. To do so, we treated the cells with either UVC light or H_2_O_2_ and monitored the monoubiquitination of PCNA by western blotting. As shown in **Figure 1A**, mUb-PCNA was not or hardly detectable in either Ramos or BL2 cells (even after long exposure) compared to non-Burkitt MRC5 and U2OS cells, which showed clear mUb regardless of treatment. These results could suggest that the monoubiquitination of PCNA in response to genotoxic stress is defective, weak or inefficient in Ramos cells. Nevertheless, monoubiquitination of PCNA is a reversible process, and its removal is catalyzed by the deubiquitinase USP1. Thus, the balance between the opposing actions of specific ubiquitin ligases and USP1 ultimately determines the ubiquitination status of PCNA. Therefore, we next asked whether the deubiquitination reaction is predominant due to abnormal expression of USP1 in Ramos and BL2 cells compared to normal B cells. To answer this question, we analyzed the expression of USP1 by western blotting in Ramos and several other Burkitt lymphoma (BL) and non-BL cell lines. To establish a comparison scale, we used tonsillar B cells, which represent physiological counterpart cells, to estimate the physiological quantity of USP1 expressed in B cells. As shown in **Figures 1B–D**, Ramos and all BL cell lines analyzed expressed high levels of USP1 compared to tonsillar B cells. Interestingly, with the exception of MCF7 cells, the expression in all other non-BL cells remained within the range of B cell physiological expression (**Figure 1C**). In addition, it should be noted that in BL cells, high USP1 levels do not depend on their EBV status since both EBV-positive (Daudi, BL16 and Raji cells) and EBV-negative (Ramos, BL2, BL1, BL29, BL74) BL cells have similar levels. Similar elevated expression of USP1 was found in human B cells derived from mantle cell lymphoma (**Figure 1D**). Together, these data suggest that the high expression/activity of USP1 in Ramos and probably most BL cell lines is responsible for the observed low mUb status of PCNA and consequently could explain the origin of the alteration in the A/T mutation pathway.

### USP1 Inhibition Increases the Half-Life of PCNA Monoubiquitination in the Ramos Cell Line

**Figure 2:**
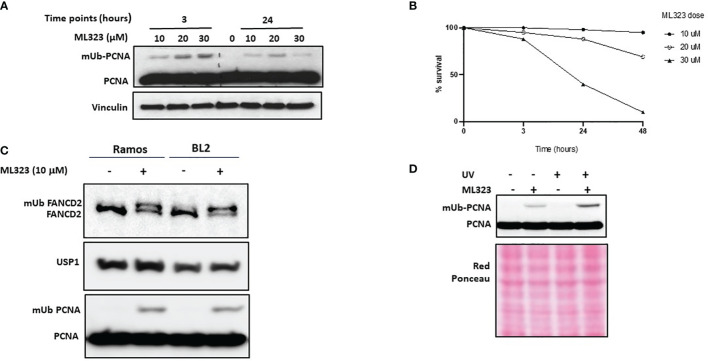
Inhibition of USP1 increases the monoubiquitination of both PCNA and FANCD2. Ramos cells were incubated with increased doses of ML323, and the monoubiquitination of PCNA and the cell toxicity of the drug at the indicated times were determined by SDS–PAGE and immunoblotting in **(A)** and using a cell survival assay in **(B)**, respectively. The data in B represent the mean of three independent experiments. **(C)** Ramos and BL2 cells were treated for two hours with 10 μM ML323, and monoubiquitination of PCNA and FANCD2 was analyzed by SDS–PAGE and immunoblotting. **(D)** Ramos cells were treated with UVC, ML323 or both, and the monoubiquitination of PCNA was analyzed as described above. Vinculin and red Ponceau were used as loading controls.

We have shown above a possible imbalance in PCNA mono-Ub/de-Ub reactions due to the elevated expression of USP1. We therefore anticipated a higher ongoing deubiquitination reaction in those cells mimics PCNA monoubiquitination deficiency. To test this hypothesis, we first treated Ramos cells for 3 to 24 hours with increasing concentrations of ML323, a selective inhibitor of USP1, and monitored both the efficacy and toxicity of the drug. As shown in **Figure 2A**, after 3 hours of treatment, we detected a dose-dependent increase in the levels of mUb-PCNA. At 24 hours, treatment with 10 μM ML323 maintained a detectable fraction of mUb-PCNA in the cells. Exposure to higher concentrations induced rapid and greater mUb-PCNA at 3 hours, but this increase was followed by a sharp decrease at 24 h, probably due to the toxicity of the drug at high concentrations (**Figure 2B**). Indeed, while more than 95% of cells remain alive in the presence of 10 μM ML323 at 48 hours, exposure to 30 μM kills 60% of the cells at 24 hours and more than 90% at 48 hours of treatment. Incubation with 20 μM killed 10% of the cells at 24 hours and 30% at 48 hours. Similar results were obtained with the BL2 cell line (data not shown). We therefore decided to use a dose of 10 μM as an effective and nontoxic concentration for the next experiments. In parallel to its role in the process of TLS, USP1 participates in the Fanconi anemia pathway through monodeubiquitination of FANCD2 during DNA interstrand crosslink lesion repair (31, 32). As expected, treatment of both Ramos and BL2 cells with ML323 led to the detection of a clear band that corresponded to mUb-PCNA and mUb-FANCD2 even in the absence of any genotoxic treatment (**Figure 2C**). Furthermore, the combination of USP1 inhibition and UVC irradiation further increased mUb-PCNA levels (**Figure 2D**). Together, these results confirm that in Ramos cells, the PCNA monoubiquitination reaction is efficient and UV-inducible. We conclude that the observed absence of mUb-PCNA in Ramos cells is the consequence of high ongoing monodeubiquitination reactions that result from higher USP1 expression and activity.

### Inhibition of PCNA Deubiquitination Increases the Rate of A/T Mutations

**Figure 3:**
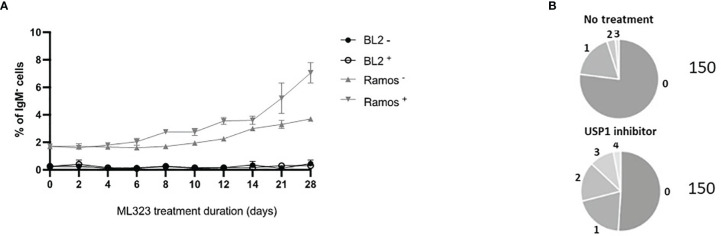
Treatment of Ramos cells, but not BL2 cells, with a USP1 inhibitor increased IgM loss compared to that of nontreated cells. **(A)** Cells were treated with 10 μM ML323 (+) or DMSO (–), and the percentage of surface IgM was measured by FACS at the indicated time points. **(B)** Sequence analysis of SHM in the amplified Ramos VH region. Relative amounts of sequences with the indicated number of mutations (from 0 to 4) are given in the pie charts. The total number of analyzed sequences is indicated in the right of each chart and corresponds to the pool of the three experiments reported in **Table 1**.

To assess whether increasing the mUb-PCNA half-life can restore the rate of A/T mutations during SHM, we treated Ramos cells with 10 μM ML323 continuously for 6 weeks to allow the cells to accumulate a sufficient number of mutations. To maintain elevated levels of mUb-PCNA throughout that period, we added fresh medium containing the drug every 2 days. Ongoing SHM in Ramos cells in culture generates diverse IgM-loss subclones without affecting cell viability due to the occurrence of stop codons, indels and frameshift mutations in the V_H_ (19). Therefore, the detection of IgM-negative cells by fluorescence-activated cell scanning (FACS) provides a quick read-out and convenient semiquantitative measure of SHM. During the 6 weeks of treatment, surface IgM was assessed by FACS (**Figure 3A**). We observed an accumulation of IgM-negative Ramos cells over time in both the presence and absence of ML323. However, USP1 inhibition further increased the percentage of IgM-negative cells. Of note, BL2 cells that do not undergo constitutive SHM do not show IgM-negative cell accumulation even in the presence of ML323. This suggests that the increase in cellular mUb-PCNA levels quantitatively participates in the processes of SHM that generate the IgM- population. At the end of treatment, the IgM- cells were FACS sorted, and VH4 segments were PCR amplified, cloned, and sequenced to appreciate whether the treatment impacts only quantitatively the process of SHM or causes a mutation pattern change or both. Interestingly, the data presented in **Table 1** show both quantitative and qualitative changes. USP1 inhibition in three independent experiments (E1-3 **Table 1**), led not only to an overall increase in unique mutation frequency in treated versus nontreated cells (0.19 versus 0.086 mut/100 bp) caused mainly by the increase of number of mutation per sequence (**Figure 3B**), but also to significant increases in the rate of A/T mutations. Indeed, while the rate of A/T mutations remained at approximately 13% (7 to 18%) for the nontreated cells, the rate increased significantly to 31% (24 to 38%) in treated cells (**Table 1**). Collectively, these results suggest that an increase in the availability of mUb-PCNA in the cell is sufficient to promote the induction of A/T mutations and further confirm that the high turnover of mUb-PCNA is responsible for the low rates of A/T mutations during SHM in Ramos cells.

**Table 1:** Somatic mutations in VH4 sequence (338 bp) from Ramos cell treated or not with USP1 inhibitor.

IgM^-^ cells(6 weeks)		Total nucleotides sequenced*	Number of mutated sequences(Unique)	Number of substitutions	Mutation frequency (per 100 bp)	Number of AT/GC mutations	% AT/GC	AT % mean value(SD)
Ramos	E1	16900	10	15	0.089	1/14	6.7/93.3	13.6 (6.219)
	E2	16900	13	16	0.095	3/13	18.75/81.25	
	E3	16900	11	13	0.077	2/11	15.4/74.6	
Ramos + ML323 (USP1i)	E1	16900	18	25	0.148	6/19	24/76	33 (7.810)
	E2	16900	25	35	0.201	13/22	37/63	
	E3	16900	30	42	0.249	16/26	38/62	

E1 means experiment number 1. E1, E2 and E3 are independent experiments. *50 clones. P= 0.0282; Two-tailed P value in unpaired t test for the AT% mean value comparison; SD. standard deviation.

### Mimicking mUb-PCNA in Ramos Cells by Ubiquitin-PCNA Fusion: Validation of the Experimental Approach

**Figure 4:**
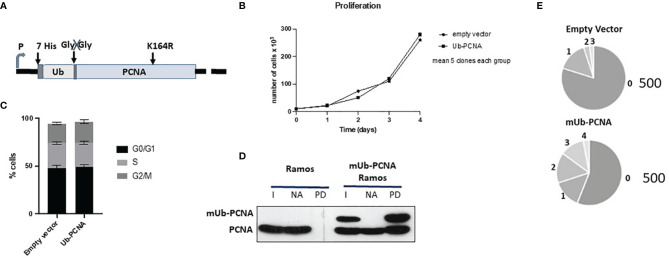
Schematic diagram of the mUb-PCNA fusion construct and validation in Ramos cells. **(A)** Schematic diagram of the mUb-PCNA fusion construct used. **(B)** Cell proliferation analysis. Cell growth was estimated by counting the viable cells on the indicated days. **(C)** Cell cycle analysis by DNA content estimation with flow cytometry and represented as histograms showing the relative percentage of cells at the indicated phases of the cell cycle. The data in B and C represent the mean of three independent experiments. **(D)** His7-UbPCNA was pulled down with nickel beads, and the fraction pulled down was analyzed by SDS–PAGE and immunoblotting using an anti-PCNA antibody. I, input; NA nonadsorbed, PD pull down. **(E)** Sequence analysis of SHM in the amplified Ramos VH region. The pie segments represent the proportion of clones that contained the specified number of mutations (from 0 to 4) indicated. The total number of analyzed sequences is indicated in the right of each chart and corresponds to the pool of the data obtained from the five clones reported in **Table 2A**.

Artificial Ub and PCNA fusion proteins have been successfully used in both yeast and mammalian cells to mimic native ubiquitinated PCNA (33–36). To avoid the use of inhibitors that could interfere with the physiological functions of the cell, we provided an exogenous modification by stably expressing mUb-PCNA fusion. The human PCNA K164R mutant sequence was used for this construction to prevent additional *in vivo* PCNA ubiquitination at the K164 residue during Ig SHM. We next added N-terminal fusion with 7x His-tagged ubiquitin to mimic PCNA-K164 monoubiquitination and to facilitate purification and analysis. Finally, to prevent its cleavage by USP1 in the cell, the C-terminal glycine-glycine residue was removed from the Ub polypeptide in the fusion construct (**Figure 4A**). Stable clones were obtained after plasmid transfection and puromycin selection. First, we performed several experiments to show that mUb-PCNA fusion expression does not perturb cell growth and participates in DNA replication as the endogenous. (i) We showed that the constitutive expression of mUb-PCNA does not modify the cell cycle and does not affect cellular proliferation (**Figures 4B, C**), indicating that mUb-PCNA fusion proteins do not affect DNA replication. (ii) We verified that the exogenous mUb-PCNA fusion protein was able to interact with endogenous PCNA to form a physiological homotrimeric ring. To this end, we used nickel beads to pull down exogenous mUb-PCNA containing a poly-His tag in the N-terminal region of ubiquitin and searched for the presence of endogenous PCNA in the pulled down fraction. As expected from previous studies (35), **Figure 4D** shows that the pulled down fraction contains similar quantities of endogenous and mUb-PCNA, consistent with the fusion proteins being able to interact equally with the untagged protein molecules. Together, these data demonstrate that USP1-resistant mUb-PCNA fusions behave similarly to the endogenous form. Therefore, Ramos cells expressing such a construct can be confidently used to study *in vitro* the mechanisms of SHM.

### Expression of Mono-Ub PCNA Fusion Protein Is Sufficient to Increase the Rate of A/T Mutations

**Figure 5:**
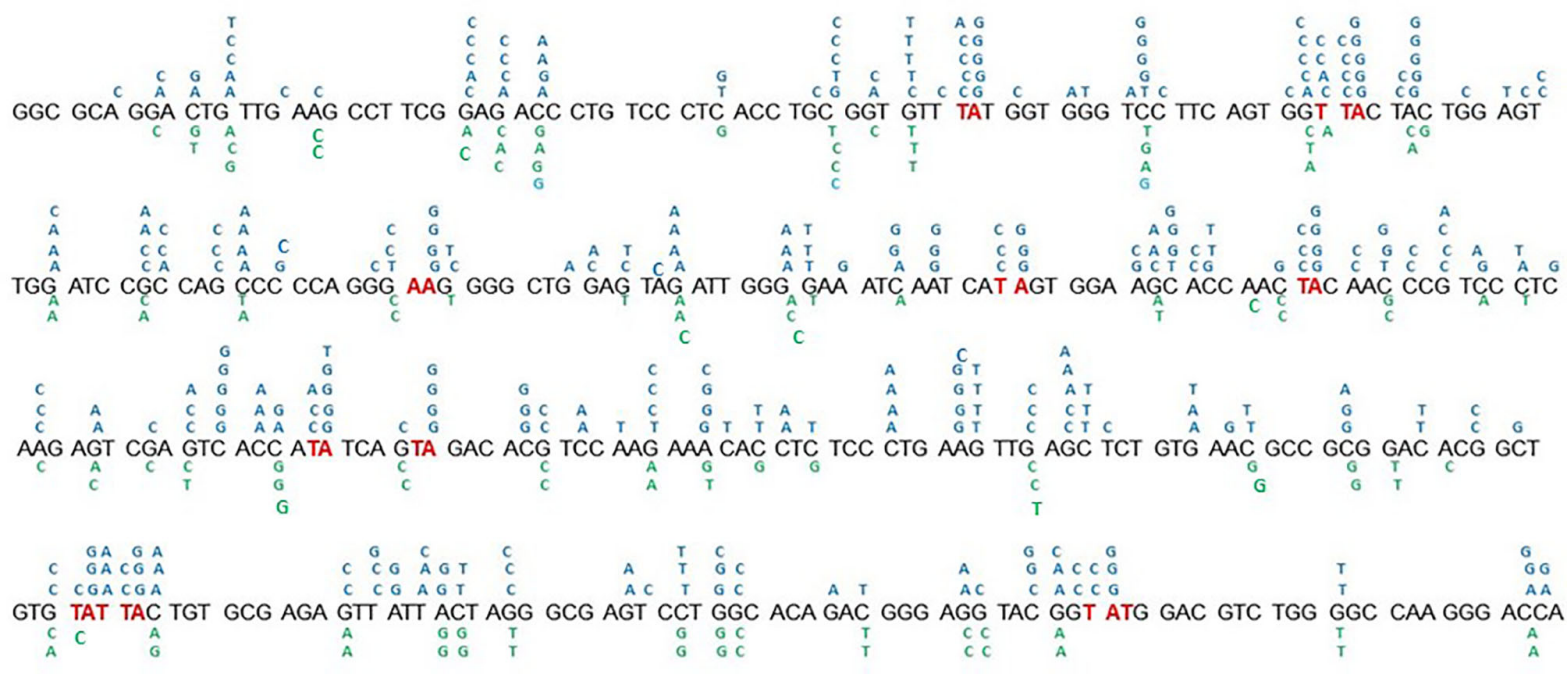
Distribution of point-mutations along the amplified Ramos VH region. Independently occurring base substitutions are indicated at each nucleotide position. The Pol h hotspots (WA/TW) targeted following the expression of mUb-PCNA are in indicated in red. The figure represent the pool of base substitution obtained from the clones indicated in **Table 2A**. The Nucleotide Substitutions in blue indicated above the Ramos VH sequence are from the 5 clones expressing mUb-PCNA and those below in green are from the five control clones.

Taking into account the aforementioned validations, we measured the impact of mUb-PCNA expression on the rate and pattern of SHM. After 6 weeks in culture, genomic DNA was extracted from several clones, and VH4 segments were PCR amplified, cloned, and sequenced. As shown in **Table 2A**, the clones expressing exogenous mUb-PCNA showed an increase of mutation frequency as seen above after the use of USP1 inhibitor, with an increase of both mutated sequences and number of mutation/sequence (**Figure 4E**). Interestingly we observed an increased rate of A/T mutations of approximately 30% (22 to 40%) compared to nonexpressing clones, which remained around 10% (5-15%). It should be noted that, as reported before, there is variability in the rate of A/T mutations in the different clones (discussed below); nevertheless, in general, the mutating clones expressing mUb-PCNA show significantly higher A/T mutation rates compared to nonexpressing clones. We next compared the mutation profile of both clones expressing and non-expressing mUb-PCNA expressing and non-expressing mUb-PCNA. The distribution of point-mutations along the amplified Ramos VH region presented in **Figure 5** showed in addition to an increase of number of mutations, differences in their base substitution characteristics. Indeed, we observed that most of the A/T mutations induced following mUb-PCNA expression (base substitution in blue) are targeted to the described polη hotspots (WA/TW). Furthermore we observed within these hotspots (red color) a clear A to G and T to C transition bias (**Figure 5**). Since the preferred mutation of Polη when copying normal DNA is the incorporation of Gs opposite Ts, thereby generating T to C and A to G transition mutations, thus strongly suggesting that these mutations are introduced by polη.

**Table 2A:** Somatic mutations in VH4 sequence (338 bp) from mUb-PCNA expressing and non-expressing Ramos clones.

IgM^-^ cells (6 weeks)		Total nucleotides sequenced*	Number of mutated sequences (unique)	Number of substitutions	Mutation frequency (per 100 bp)	Number of AT/GC mutations	% AT/GC	AT % mean value(SD)
Ramos + Ub-PCNA	R4.16	33800	55	90	0.266	36/54	40/60	30.38 (6.861)
	R2.4.6	33800	52	79	0.234	27/52	34/66	
	R1.16	33800	40	70	0.207	20/50	28.5/71.5	
	R3.2	33800	37	62	0.183	17/43	27.4/72.6	
	R3.7	33800	35	50	0.148	11/39	22/78	
Ramos control (empty vector)	R13.5	33800	17	20	0.060	1/19	5/95	9.18 (3.571)
	R2.4.8	33800	21	24	0.071	3/24	12.5/87.5	
	R1.12	33800	20	25	0.074	3/23	13/87	
	R2.5	33800	26	30	0.089	2/28	6.7/93.3	
	R3.8	33800	19	25	0.074	2/23	8.7/91.3	

*100 clones.

P= 0.0003 (two-tailed P value, unpaired t test) for the AT% mean value comparison; SD, standard deviation.

**Table 2B:** Somatic mutations in Constant μ-region sequence (550 bp) from mUb-PCNA expressing Ramos clones after six weeks and three months in culture.

IgM^-^ cells		Total nucleotides sequenced* (x10^3^)	Number of mutated sequences (unique)	Number of substitutions	Mutation frequency (per 100 bp)(x10^-3^)	Number of AT/GC mutations	% AT/GC	AT % mean value
Ramos + Ub-PCNA 6 weeks	R4.16	55	0	0				
	R2.4.6	55	1	1	1.8	0/1		
	R1.16	55	1	1	1.8	1/0		
	R3.2	55	0	0				
	R3.7	55	0	0				
Ramos + Ub-PCNA 3 months	R4.16	55	1	1	1.8	0/1		
	R2.4.6	55	1	1	1.8	1/0		
	R1.16	55	2	2	3.6	0/2		
	R3.2	55	0	0				
	R3.7	55	1	2	3.6	0/2		

*100 clones.

Together, these results indicate that the expression of stable mUb-PCNA is sufficient to retrieve the A/T mutagenesis pathway in Ramos cells.

### Induction of A/T Mutations Is AID- and POLH-Dependent in Ramos-Ub Cells

**Figure 6:**
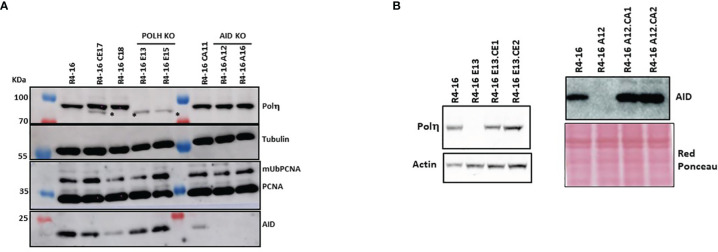
Inactivation and restoration of polη and AID in the R4-16 clone. CRISPR/Cas9 was used to inactivate and gRNA editing were used to inactivate polη or AID. **(A)** The absence of the corresponding proteins was confirmed in two selected clones by immunoblot analysis. **(B)** KO clones were reconstituted by stable expression of POLH or AID cDNA. Left panel: R4-16E13-CE1 and R4-16E13-CE2 are two clones derived from the human polη deficient clone R4-16E13 after transfection with polη-expressing vector. Right panel: R4-16A12-CA1 R4-16A12-CA2 are two clones derived from the AID-deficient clone R4-16A12 after transfection with the human AID-expressing vector. Actin and red Ponceau staining were used as loading controls. *Nonspecific band.

Since polη is the major A/T mutator in both mice and humans during SHM, we asked whether the induced A/T mutations following the expression of mUb-PCNA are generated through a genuine SHM process that requires AID activity and are polη-dependent. To this end, we inactivated the genes encoding AID (*AICDA*) or polη (*POLH*) using CRISPR/Cas9 technology in the Ramos R4-16 subclone. It should be noted that, among the clones with increased rates of A/T mutations we chose clone R4-16 to continue our investigations, for two main reasons: (i) it displays a higher rate of A/T mutations and (ii) it is stable and retained ongoing SHM when maintained in culture for up to 3 months. The absence of AID and POLH protein expression was validated by immunoblotting (**Figure 6A**). POLH-deficient clones were grown for 6 weeks, and the expressed V(D)J was amplified and sequenced. **Table 3** shows that in the polη-deficient clones, A/T mutations decreased to the level of the wild-type Ramos cell population. Induction of A/T mutations was restored upon re-expression of human *POLH* cDNA in these clones to frequencies comparable to those observed in the Ramos mUb-PCNA parental clone (**Figure 6B**, left and **Table 3**). In addition, inactivation of *AID* completely abolished both A/T and G/C mutations. Similarly, the re-expression of *AID* restored the mutations to the level of the parental clone (**Figure 6B**, right and **Table 3**). Altogether, these results confirm that the A/T mutations induced following mUb-PCNA expression result from a genuine immunoglobulin V gene diversification mechanism initiated by AID and generated by polη activity.

**Table 3:** Somatic mutations in VH4 sequence (338 bp) from cultured Ramos clones knocked-out for *POLH* or *AID* in the mUb-PCNA background.

IgM^-^ cells(6 weeks)		Total nucleotides sequenced*	Number of mutated sequences(Unique)	Number of substitutions	Mutation frequency (per 100 bp)	Number of AT/GC mutations	% AT/GC	AT % mean value
Ramos + Ub-PCNA *POLH* KO	R4.16-E13	33800	18	24	0.071	4/20	16.7/83.3	17.25
	R4.16-E15	33800	23	28	0.083	5/23	17.8/82.2	
Ramos control (empty vector)	R4.16-E17	33800	53	80	0.237	33/47	41/59	38.8
Ramos + mUb-PCNA *POLH* KO + hPOLH	R4.16-E13 CE 1	33800	42	51	0.151	16/35	31.4/68.6	32.65
	R4.16-E13 CE 2	33800	45	62	0.183	21/41	33.9/66.1	
Ramos + mUb-PCNA *AID* KO	R4.16-A12	33800	2	2	0.006	1/1	ND	ND
	R4.16-A16	33800	1	2	0.006	0/2	ND	
Ramos control (empty vector)	R4.16-A11	33800	50	82	0.243	30/52	36.6/63.4	38.8
Ramos + mUb-PCNA *AID* KO + hAID	R4.16-A12 CA1	33800	21	29	0.085	8/21	27.6/72.4	31.65
	R4.16-A12 CA2	33800	26	28	0.083	10/28	35.7/64.3	

*100 clones; ND, none detected; CE complemented with exogenous pol η CA complemented with exogenous AID.

### Absence of Genome Wide Mutagenesis

Since mUb-PCNA preferentially recruits low-fidelity TLS polymerase, we wondered whether the constitutive expression of mUb-PCNA could also promote mutations elsewhere in the genome. To answer this question, on the one hand, we sequenced the μ-constant region known not to be targeted by physiological SHM and on the other hand estimated the mutation rate at the hypoxanthine-guanine phosphoribosyl transferase (*HPRT*) locus^17^. As shown in **Table 2B**, mutations did not accumulate in Cμ even after 3 months in culture. Furthermore, we found a similar mutation rate at the HPRT locus, approximately 1.5 to 2.1 X 10^-7^ mutations per locus and per generation, determined by fluctuation analysis of resistance to 6-thioguanine in both clones expressing or not expressing mUb-PCNA (data not shown). Collectively, these results allow us to conclude that the constitutive expression of mUb-PCNA in Ramos cells induces and targets A/T mutations to the Ig locus and does not induce a general mutator phenotype.

## Discussion

We report the establishment of a cell line that can be used to address *in vitro* the mechanism of A/T mutations during the process of somatic hypermutation of Ig genes. The Ramos Burkitt cell line has been widely used to study the mechanism of SHM *in vitro* (19, 37, 38). Ramos cells exhibit most of the features of SHM *in vivo* except that the spectrum of mutations displays a deficiency in A/T mutations. Indeed, 85 to 90% of the mutations are at G/C residues compared to *in vivo*, where mutations are distributed equally on the G/C and A/T bases. We have demonstrated in this study that the paucity of A/T mutations in Ramos cells is due to the high activity of USP1, which favors the deubiquitination of mUb-PCNA. To bypass this limitation, we either selectively inhibited the activity of USP1 or constitutively expressed mono-Ub PCNA. In both cases, we increased the rate of A/T mutations at the expressed Vh gene. Finally, we showed that these mutations are genuine SHM, depending on both AID and polη. The SHM mechanism depends on the deaminase activity of AID, which converts deoxycytidines (dCs) to deoxyuridines (Us) in both strands of DNA. The processing of the U/G mismatch, thus created by several DNA damage responses, is generated at the site of the leading to G/C mutations, both transitions and transversions, and A/T mutations around the lesion, resulting in similar rates of mutation at both A/T and G/C bp. Although the current knowledge of DNA repair mechanisms in mammalian cells can explain the spectrum of G/C mutations following high rate deamination of dC in S phase of the cell cycle regardless of its origin, introduction of mutations at the A/T bases that occurs mainly in G1 phase of activated B cells is more challenging. Furthermore, this pathway is restricted to the diversification of Ig genes during the maturation of the immune response, it results from diverted DNA repair factors and remains poorly understood to date. Investigations to decipher the underlying molecular mechanisms are limited by the absence of a cellular model able to faithfully reproduce the mechanism of both G/C and A/T base mutagenesis.

In this report, we used a Ramos cell line that constitutively diversifies its rearranged immunoglobulin V gene during *in vitro* culture. This ongoing process does not require the help of activated T cells, added cytokines, or B cell antigen receptor signaling. However, these cells mainly use the G/C mutation pathways to diversify their Igs. The absence or low rate of the A/T mutagenesis process led to the neglect of this model. In B cells, the U/G mismatch is recognized by Msh2-Msh6 and recruits exonuclease I (ExoI), which creates a long single-stranded gap. Then, polη is recruited *via* mUb-PCNA, leading to error-prone gap filling (7, 11, 39). The specific activity of polη when copying undamaged DNA, which copies Ts with very low fidelity, generates the majority of mutations at A/T bases away from the AID-modified C base. We therefore postulated that either one or several components of the A/T pathway are missing or malfunctioning in Ramos cells.

We showed that the paucity of A/T mutations is due to the high activity of ubiquitin-specific protease 1, which is responsible for the short half-life of mUb-PCNA. We have shown that the inhibition of USP1 activity using ML123, a potent and specific inhibitor, increases the fraction of cellular mUb-PCNA in Ramos cells, which in turn favors an increase in the rate of A/T mutations at the Ig locus. These data indicate that although the reaction of PCNA ubiquitination is intact in Ramos cells, its deubiquitination rate is higher, greatly shortening its half-life and thus limiting its participation in the A/T mutagenesis pathway. Of note, elevated expression of USP1 has been reported in several human cancers, including osteosarcoma, non-small-cell lung cancer, and breast and colorectal cancers (40–42). This study reports for the first time elevated expression of USP1 protein in both Burkitt lymphoma and mantle cell lymphoma.

Since PCNA is not the only USP1 target in mammalian cells and to avoid any nonspecific or off-target effects, we substituted the use of USP1i, which served as proof of principle, by stably expressing the mUb-PCNA fusion in Ramos cells. Indeed, artificial Ub and PCNA fusion proteins have been successfully validated and used in both yeast and mammalian cells to mimic native ubiquitinated PCNA and bypass the requirement for PCNA monoubiquitination in response to UV DNA damage (34–36). We showed that the stable expression of USP1-resistant mUb-PCNA in Ramos cells resulted in an increase in the rate of Ig A/T mutations without affecting cell proliferation or the cell cycle profile. mUb-PCNA pull-down experiments in Ramos show that the latter interacts with endogenous PCNA and participates in the formation of the sliding ring, suggesting that this fusion protein fulfills cellular function(s) similar to endogenous monoubiquitinated PCNA. This result is in agreement with previous reports showing (i) that yeast cells can survive mUb-PCNA fusion in the absence of endogenous PCNA and (ii) that in mammalian cells, mUb-PCNA fusion can be loaded onto DNA and is able to protect host cells from UV-induced DNA damage, with characteristic TLS activities, thus mimicking endogenous PCNA monoubiquitination (36). Of note the analysis of the mutation pattern show that beside the high increase of A/T mutations, we observed also a small increase of G/C mutations. This latter was not expected since *in vivo* the analysis of SHM in PCNAK164R expressing cells show mainly an impact on A/T mutations. Although we cannot propose any obvious explanation, we observed within the G/C mutation an increase of C to G and G to C (107 out of a total of 238 G/C mutations in the presence of mUb-PCNA (45%) versus 57/137 (39.4% in its absence); this could be partly attributed to higher recruitment of Rev1 in the presence of constitutive expression of USP1 resistant mUb-PCNA. The permanent availability of USP1-resistant mUb-PCNA into the cells may provide mUb-PCNA more time, although to a lesser extent, to recruit other TLS that are normally not recruited under physiological conditions where PCNA is continuously ubiquitinated and de-ubiquitinated.

Importantly, we demonstrate that the introduced A/T mutations in Ramos cells expressing mUb-PCNA fusion are genuine SHM for several reasons: i) are mainly targeted to WA hotspots with a strong A to G and T to C transition bias which is strictly attributable to the enzymatic specificity of pol η ii) they depend on the A/T pathway master genes *AID* and *POLH*. Importantly we found that both A/T and G/C mutations are absent in the AID-deficient clones, and polη deficiency leads to low A/T mutation even in the presence of constitutive expression of mUb-PCNA. iii) they are strictly targeted to the expressed V gene. Indeed, no mutations were detected in the constant μ-region, and the constitutive expression of mUb-PCNA does not lead to an overall increase in genome-wide mutagenesis as assessed by the rate of mutations at the *HPRT* locus. These results are in agreement with previous studies showing that the expression of mUb-PCNA fusion does not cause an increase of spontaneous mutations (34, 35)

Although the mUb-PCNA fusion has been used to study the mechanism of DNA damage tolerance in response to replication-blocking lesions induced by a variety of genotoxic agents (in human and yeast cells), this study is the first to address its role in the specific recruitment of polη during the mechanism of Ig A/T base mutations that occur in the absence of any DNA lesion, by DNA gap filling in instead of bypass and likely independently of replication stress or fork stalling signaling.

The Ramos BL cell line used in the laboratory was first described as a population that constitutively mutates its rearranged V(D)J region at a rate of 2 to 3x10^-5^ mutations/bp/generation (19). In this study, we found that the expression of mUb-PCNA fusion modifies both the pattern and the rate of mutations. Similar to what has been shown previously (43), the analysis of several individual subclones expressing mUb-PCNA isolated from the original population after transfection revealed the presence of 15 to 20% nonmutated clones and that almost 35 to 50% of clones progressively lost the ability to mutate their V region over time when maintained in culture (1 to 3 months) regardless of the expression of AID and/or mUb-PCNA. The remaining 20 to 30% display variable ongoing mutation rates ranging from 2 x 10^-5^ to 6 x 10^-4^ mutations/bp/generation. Despite this finding that suggests a clonal variation and instability of Ramos clones that should be taken into account when working with this cell line, the expression of mUb-PCNA increased the mutation rates of A/T bases in all clones that continue to undergo SHM when maintained in culture. Finally, we observed that in the clones with the highest mutation rates, the A/T mutations did not reach 45 to 50%, as expected. This could be partially attributed to the decrease in the number of AID hot spots in the V(D)J sequence over time compared to the corresponding germline sequence due to the ongoing SMH. It would be interesting to use the recombinase-mediated cassette exchange system established by MD Scharff’s lab (38) to exchange endogenous V(D)J in Ramos cells expressing mUb-PCNA, analyze the rate and pattern of A/T and C/G mutations and compare them to the *in vivo* pattern.

In summary, we have established and validated a cell model with few limitations that reproduces the full process of somatic hypermutation of Ig genes *in vitro* and can be used to answer questions concerning the A/T mutation pathway that cannot be addressed otherwise. For example, how, in activated B cells, ncMMR activity is targeted to Ig loci and becomes active in G1 phase of the cell cycle? While mUb-PCNA is required for pol η recruitment for TLS in response to replication-blocking UV lesions, it remains unclear how polη is selectively recruited to fill in the gap created by ncMMR activity. How are the other DNA polymerases, both error-free and error-prone, actively excluded? We expect that these questions and others will be addressed with the use of this *in vitro* cellular model.

## Data Availability Statement

The original contributions presented in the study are included in the article/supplementary material. Further inquiries can be directed to the corresponding author.

## Author Contributions

SA designed and supervised the study. LL, DB, MG, MM, ZK, and ED performed experiments. SA, LL, DB, and AS analyzed and curated the data. SA, LL, ED, and AS participated in the writing of the paper. All authors read, reviewed, and approved the final manuscript.

## Funding

This study was funded by La Ligue Nationale Contre le Cancer (Equipe labellisée EL2018_Kannouche) (SA); Gustave Roussy Cancer Campus, the Centre national de la recherche scientifique (CNRS). (ED) was supported by grants from Institut National du Cancer (INCa PLBIO16-011). Association des Enfants de La Lune (Bellegarde-sur-Valserine, France) (AS). LL received a 1-year funding from the Coordenação de Aperfeiçoamento de Pessoal de Nível Superior (CAPES, Brasília, DF, Brazil).

## Conflict of Interest

The authors declare that the research was conducted in the absence of any commercial or financial relationships that could be construed as a potential conflict of interest.

## Publisher’s Note

All claims expressed in this article are solely those of the authors and do not necessarily represent those of their affiliated organizations, or those of the publisher, the editors and the reviewers. Any product that may be evaluated in this article, or claim that may be made by its manufacturer, is not guaranteed or endorsed by the publisher.

## References

[1] Di Noia JM Neuberger MS. Molecular Mechanisms of Antibody Somatic Hypermutation. Annu Rev Biochem (2007) 76:1–22. doi: 10.1146/annurev.biochem.76.061705.090740 17328676

[2] Pilzecker B Jacobs H. Mutating for Good: DNA Damage Responses During Somatic Hypermutation. Front Immunol (2019) 10:438. doi: 10.3389/fimmu.2019.00438 30915081 PMC6423074

[3] Rogier M Moritz J Robert I Lescale C Heyer V Abello A. Fam72a Enforces Error-Prone DNA Repair During Antibody Diversification. Nat (2021) 600(7888):329–33. doi: 10.1038/s41586-021-04093-y 34819671

[4] Feng Y Li C Stewart JA Barbulescu P Seija Desivo N Álvarez-Quilón A. FAM72A Antagonizes UNG2 to Promote Mutagenic Repair During Antibody Maturation. Nat (2021) 600(7888):324–8. doi: 10.1038/s41586-021-04144-4 PMC9425297 34819670

[5] Zeng X Winter DB Kasmer C Kraemer KH Lehmann AR Gearhart PJ. DNA Polymerase Eta is an A-T Mutator in Somatic Hypermutation of Immunoglobulin Variable Genes. Nat Immunol (2001) 2(6):537–41. doi: 10.1038/88740 11376341

[6] Delbos F De Smet A Faili A Aoufouchi S Weill J-C Reynaud C-A. Contribution of DNA Polymerase Eta to Immunoglobulin Gene Hypermutation in the Mouse. J Exp Med (2005) 201(8):1191–6. doi: 10.1084/jem.20050292 PMC2213152 15824086

[7] Delbos F Aoufouchi S Faili A Weill J-C Reynaud C-A. DNA Polymerase Eta is the Sole Contributor of a/T Modifications During Immunoglobulin Gene Hypermutation in the Mouse. J Exp Med (2007) 204(1):17–23. doi: 10.1084/jem.20062131 17190840 PMC2118439

[8] Jiricny J. Postreplicative Mismatch Repair. Cold Spring Harb Perspect Biol (2013) 5(4):a012633. doi: 10.1101/cshperspect.a012633 23545421 PMC3683899

[9] Iyer RR Pluciennik A Burdett V Modrich PL. DNA Mismatch Repair: Functions and Mechanisms. Chem Rev (2006) 106(2):302–23. doi: 10.1021/cr0404794 16464007

[10] Goellner EM Putnam CD Kolodner RD. Exonuclease 1-Dependent and Independent Mismatch Repair. DNA Repair (Amst) (2015) 32:24–32. doi: 10.1016/j.dnarep.2015.04.010 25956862 PMC4522362

[11] Langerak P Nygren AOH Krijger PHL van den Berk PCM Jacobs H. A/T Mutagenesis in Hypermutated Immunoglobulin Genes Strongly Depends on PCNAK164 Modification. J Exp Med (2007) 204(8):1989–98. doi: 10.1084/jem.20070902 PMC2118671 17664295

[12] Fox JT Lee K Myung K. Dynamic Regulation of PCNA Ubiquitylation/Deubiquitylation. FEBS Lett (2011) 585(18):2780–5. doi: 10.1016/j.febslet.2011.05.053 PMC3172383 21640107

[13] Kannouche PL Lehmann AR. Ubiquitination of PCNA and the Polymerase Switch in Human Cells. Cell Cycle (Georgetown Tex) (2004) 3(8):1011–3. doi: 10.4161/cc.3.8.1074 15280666

[14] Moldovan G-L Pfander B Jentsch S. PCNA, the Maestro of the Replication Fork. Cell (2007) 129(4):665–79. doi: 10.1016/j.cell.2007.05.003 17512402

[15] Notenboom V Hibbert RG van Rossum-Fikkert SE Olsen JV Mann M Sixma TK. Functional Characterization of Rad18 Domains for Rad6, Ubiquitin, DNA Binding and PCNA Modification. Nucleic Acids Res (2007) 35(17):5819–30. doi: 10.1093/nar/gkm615 PMC2034485 17720710

[16] Roa S Avdievich E Peled JU MacCarthy T Werling U Kuang FL. Ubiquitylated PCNA Plays a Role in Somatic Hypermutation and Class-Switch Recombination and Is Required for Meiotic Progression. PNAS (2008) 105(42):16248–53. doi: 10.1073/pnas.0808182105 PMC2571010 18854411

[17] Langerak P Krijger PHL Heideman MR van den Berk PCM Jacobs H. Somatic Hypermutation of Immunoglobulin Genes: Lessons From Proliferating Cell Nuclear Antigenk164r Mutant Mice. Philos Trans R Soc Lond B Biol Sci (2009) 364(1517):621–9. doi: 10.1098/rstb.2008.0223 PMC2660925 19008189

[18] Denépoux S Razanajaona D Blanchard D Meffre G Capra JD Banchereau J. Induction of Somatic Mutation in a Human B Cell Line *In Vitro* . Immunity (1997) 6(1):35–46. doi: 10.1016/S1074-7613(00)80240-X 9052835

[19] Sale JE Neuberger MS. TdT-Accessible Breaks Are Scattered Over the Immunoglobulin V Domain in a Constitutively Hypermutating B Cell Line. Immun (1998) 9(6):859–69. doi: 10.1016/S1074-7613(00)80651-2 9881976

[20] Zan H Cerutti A Dramitinos P Schaffer A Li Z Casali P. Induction of Ig Somatic Hypermutation and Class Switching in a Human Monoclonal IgM+ IgD+ B Cell Line *In Vitro*: Definition of the Requirements and Modalities of Hypermutation. J Immunol (1999) 162(6):3437–47. PMC4623562 10092799

[21] Faili A Aoufouchi S Guéranger Q Zober C Léon A Bertocci B. AID-Dependent Somatic Hypermutation Occurs as a DNA Single-Strand Event in the BL2 Cell Line. Nat Immunol (2002) 3(9):815–21. doi: 10.1038/ni826 12145648

[22] Faili A Aoufouchi S Flatter E Guéranger Q Reynaud C-A Weill J-C. Induction of Somatic Hypermutation in Immunoglobulin Genes Is Dependent on DNA Polymerase Iota. Nature (2002) 419(6910):944–7. doi: 10.1038/nature01117 12410315

[23] Klein U Klein G Ehlin-Henriksson B Rajewsky K Küppers R. Burkitt’s Lymphoma is a Malignancy of Mature B Cells Expressing Somatically Mutated V Region Genes. Mol Med (1995) 1(5):495–505. doi: 10.1007/BF03401587 8529116 PMC2229966

[24] Xiao Z Ray M Jiang C Clark AB Rogozin IB Diaz M. Known Components of the Immunoglobulin A:T Mutational Machinery are Intact in Burkitt Lymphoma Cell Lines With G:C Bias. Mol Immunol (2007) 44(10):2659–66. doi: 10.1016/j.molimm.2006.12.006 PMC1868521 17240451

[25] Kannouche P Broughton BC Volker M Hanaoka F Mullenders LH Lehmann AR. Domain Structure, Localization, and Function of DNA Polymerase Eta, Defective in Xeroderma Pigmentosum Variant Cells. Genes Dev (2001) 15(2):158–72. doi: 10.1101/gad.187501 PMC312610 11157773

[26] Helm M Riedl S AB Gollner K Gollner U Jérôme V Freitag R. Isolation of Primary Human B Lymphocytes From Tonsils Compared to Blood as Alternative Source for Ex Vivo Application. J Chromatogr B Analyt Technol BioMed Life Sci (2021) 1179:122853. doi: 10.1016/j.jchromb.2021.122853 34325309

[27] Despras E Sittewelle M Pouvelle C Delrieu N Cordonnier AM Kannouche PL. Rad18-Dependent SUMOylation of Human Specialized DNA Polymerase Eta is Required to Prevent Under-Replicated DNA. Nat Commun (2016) 7:13326. doi: 10.1038/ncomms13326 27811911 PMC5097173

[28] Harris RS Croom-Carter DS Rickinson AB Neuberger MS. Epstein-Barr Virus and the Somatic Hypermutation of Immunoglobulin Genes in Burkitt’s Lymphoma Cells. J Virol (2001) 75(21):10488–92. doi: 10.1128/JVI.75.21.10488-10492.2001 PMC114624 11581418

[29] Poltoratsky V Woo CJ Tippin B Martin A Goodman MF Scharff MD. Expression of Error-Prone Polymerases in BL2 Cells Activated for Ig Somatic Hypermutation. Proc Natl Acad Sci USA (2001) 98(14):7976–81. doi: 10.1073/pnas.141222198 PMC35453 11427727

[30] Zlatanou A Despras E Braz-Petta T Boubakour-Azzouz I Pouvelle C Stewart GS. The Hmsh2-Hmsh6 Complex Acts in Concert With Monoubiquitinated PCNA and Pol η in Response to Oxidative DNA Damage in Human Cells. Mol Cell (2011) 43(4):649–62. doi: 10.1016/j.molcel.2011.06.023 21855803

[31] Nijman SMB Huang TT Dirac AMG Brummelkamp TR Kerkhoven RM D’Andrea AD. The Deubiquitinating Enzyme USP1 Regulates the Fanconi Anemia Pathway. Mol Cell (2005) 17(3):331–9. doi: 10.1016/j.molcel.2005.01.008 15694335

[32] Liang F Miller AS Longerich S Tang C Maranon D Williamson EA. DNA Requirement in FANCD2 Deubiquitination by USP1-UAF1-RAD51AP1 in the Fanconi Anemia DNA Damage Response. Nat Commun (2019) 10(1):2849. doi: 10.1038/s41467-019-10408-5 31253762 PMC6599204

[33] Gervai JZ Gálicza J Szeltner Z Zámborszky J Szüts D. A Genetic Study Based on PCNA-Ubiquitin Fusions Reveals No Requirement for PCNA Polyubiquitylation in DNA Damage Tolerance. DNA Repair (Amst) (2017) 54:46–54. doi: 10.1016/j.dnarep.2017.04.003 28458162

[34] Pastushok L Hanna M Xiao W. Constitutive Fusion of Ubiquitin to PCNA Provides DNA Damage Tolerance Independent of Translesion Polymerase Activities. Nucleic Acids Res (2010) 38(15):5047–58. doi: 10.1093/nar/gkq239 PMC2926605 20385585

[35] Ramasubramanyan S Coulon S Fuchs RP Lehmann AR Green CM. Ubiquitin-PCNA Fusion as a Mimic for Mono-Ubiquitinated PCNA in Schizosaccharomyces Pombe. DNA Repair (2010) 9(7):777–84. doi: 10.1016/j.dnarep.2010.03.015 20452294

[36] Qin Z Lu M Xu X Hanna M Shiomi N Xiao W. DNA-Damage Tolerance Mediated by PCNA•Ub Fusions in Human Cells is Dependent on Rev1 But Not Polη. Nucleic Acids Res (2013) 41(15):7356–69. doi: 10.1093/nar/gkt542 PMC3753651 23761444

[37] Upton DC Unniraman S. Assessing Somatic Hypermutation in Ramos B Cells After Overexpression or Knockdown of Specific Genes. J Vis Exp (2011) 57):e3573. doi: 10.3791/3573 PMC3308632 22083360

[38] Baughn LB Kalis SL MacCarthy T Wei L Fan M Bergman A. Recombinase-Mediated Cassette Exchange as a Novel Method To Study Somatic Hypermutation in Ramos Cells. mBio (2011) 2(5):e00186-11. doi: 10.1128/mBio.00186-11 21990614 PMC3190358

[39] Zivojnovic M Delbos F Girelli Zubani G Julé A Alcais A Weill J-C. Somatic Hypermutation at a/T-Rich Oligonucleotide Substrates Shows Different Strand Polarities in Ung-Deficient or -Proficient Backgrounds. Mol Cell Biol (2014) 34(12):2176–87. doi: 10.1128/MCB.01452-13 PMC4054293 24710273

[40] Williams SA Maecker HL French DM Liu J Gregg A Silverstein LB. USP1 Deubiquitinates ID Proteins to Preserve a Mesenchymal Stem Cell Program in Osteosarcoma. Cell (2011) 146(6):918–30. doi: 10.1016/j.cell.2011.07.040 21925315

[41] Liu Y Luo X Hu H Wang R Sun Y Zeng R. Integrative Proteomics and Tissue Microarray Profiling Indicate the Association Between Overexpressed Serum Proteins and Non-Small Cell Lung Cancer. PloS One (2012) 7(12):e51748. doi: 10.1371/journal.pone.0051748 23284758 PMC3526638

[42] Lee J-K Chang N Yoon Y Yang H Cho H Kim E. USP1 Targeting Impedes GBM Growth by Inhibiting Stem Cell Maintenance and Radioresistance. Neuro Oncol (2016) 18(1):37–47. doi: 10.1093/neuonc/nov091 26032834 PMC4677407

[43] Zhang W Bardwell PD Woo CJ Poltoratsky V Scharff MD Martin A. Clonal Instability of V Region Hypermutation in the Ramos Burkitt’s Lymphoma Cell Line. Int Immunol (2001) 13(9):1175–84. doi: 10.1093/intimm/13.9.1175 11526098

