# Corrigendum: Expression of constitutive fusion of ubiquitin to PCNA restores the level of immunoglobulin A/T mutations during somatic hypermutation in the Ramos cell line

**DOI:** 10.3389/fimmu.2024.1419493

**Published:** 2024-05-22

**Authors:** Leticia K. Lerner, Dorine Bonte, Morwenna Le Guillou, Mahwish Mian Mohammad, Zeinab Kasraian, Alain Sarasin, Emmanuelle Despras, Said Aoufouchi

**Affiliations:** ^1^Centre National de la Recherche Scientifique UMR 9019, B Cell and Genome Plasticity Team, Villejuif, France; ^2^Gustave Roussy, Villejuif, France; ^3^Université Paris-Saclay, Orsay, France; ^4^Department of Microbiology, Institute of Biomedical Sciences, University of São Paulo, São Paulo, Brazil; ^5^Sorbonne Université, Paris, France

**Keywords:** immunoglobulin somatic hypermutation, PCNA monoubiquitination, Ramos B cell line, USP1 inhibition, A/T mutation pathway

In the published article, there was an error in **Figure 5** as published. The published version contains typing errors. The corrected **Figure 5** and its caption appear below.

**Figure 5:**
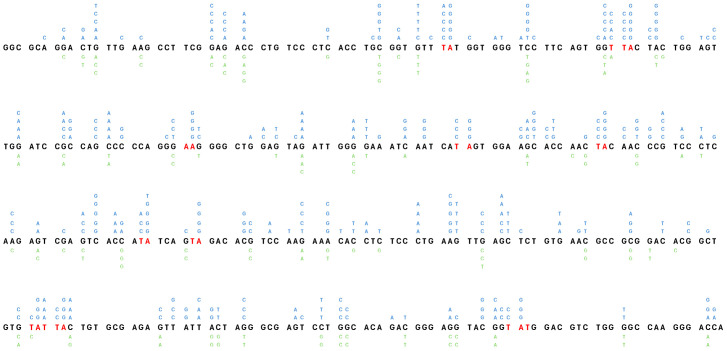
Distribution of point-mutations along the amplified Ramos VH region. Independently occurring base substitutions are indicated at each nucleotide position. The POLH hotspots (WA/TW) targeted following the expression of mUb-PCNA are in indicated in red. The figure represent the pool of base substitution obtained from the clones indicated in Table 2A. The Nucleotide Substitutions in blue indicated above the Ramos VH sequence are from the 5 clones expressing mUb-PCNA and those below in green are from the five control clones.

The authors apologize for this error and state that this does not change the scientific conclusions of the article in any way. The original article has been updated.

